# Iatrogenic Kaposi Sarcoma in an HIV‐Negative Patient With Immune Thrombocytopenic Purpura Treated With Rituximab and Corticosteroids: A Fatal Extracutaneous Presentation

**DOI:** 10.1002/cnr2.70560

**Published:** 2026-04-23

**Authors:** Rami Khader, Mohammed Yousef Awad, Ayman Zebda, Mousa Almasalma, Ro'a Draidi, Esraa Elhakim, Mohammad Bdair, Ameed Khader

**Affiliations:** ^1^ Faculty of Medicine Mansoura University Mansoura Egypt; ^2^ Department of Medicine Faculty of Medicine and Health Sciences, An‐Najah National University Nablus Palestine; ^3^ Medical Oncology Unit, Oncology Center Mansoura University Mansoura Egypt; ^4^ Department of Medicine Alexandria University Alexandria Egypt

**Keywords:** corticosteroids, HHV‐8, HIV‐negative, iatrogenic, immune thrombocytopenic purpura, Kaposi sarcoma, pulmonary Kaposi sarcoma, rituximab

## Abstract

**Background:**

Kaposi sarcoma (KS) is a Kaposi sarcoma, an Human herpesvirus 8 infection–associated endothelial neoplasm that typically arises in the setting of immune deficiency. Iatrogenic KS in HIV‐negative patients treated for Immune thrombocytopenic purpura is rare, particularly in those receiving immunosuppressive therapies such as rituximab and corticosteroids. These cases may present without characteristic cutaneous lesions, making early diagnosis challenging.

**Case Presentation:**

We report a 43‐year‐old man with immune thrombocytopenic purpura in whom rituximab was re‐initiated due to recurrent thrombocytopenia while receiving corticosteroids and eltrombopag. Following the third weekly dose, he developed progressive cough and dyspnea over one month, accompanied by severe hypoxemia. Clinical evaluation revealed a buccal mucosal lesion and generalized lymphadenopathy. Computed tomography demonstrated extensive nodal involvement with bilateral pleural effusions, ground‐glass opacities, patchy consolidation, interstitial thickening, and pulmonary nodules. Biopsies obtained from the buccal lesion and an axillary lymph node confirmed Kaposi sarcoma with positive immunohistochemical staining for Human herpesvirus 8 infection; HIV serology was negative. Treatment with weekly paclitaxel led to initial clinical improvement. However, the patient subsequently deteriorated due to pulmonary hemorrhage secondary to underlying ITP and died shortly after admission to the intensive care unit.

**Conclusion:**

This case highlights that Kaposi sarcoma should be considered in immunosuppressed but HIV‐negative patients presenting with unexplained respiratory or mucosal symptoms and lymphadenopathy, even in the absence of skin lesions. Early histological confirmation is essential to avoid diagnostic delay and to guide appropriate management.

## 1 Introduction

Kaposi sarcoma (KS) is a rare endothelial angioproliferative tumor closely related to infection with human herpesvirus 8 (HHV‐8) [1]. It was first described in 1872 by Moritz Kaposi [2], and is now classified into four subtypes: classic KS, most commonly affecting elderly Mediterranean or Eastern European people; endemic KS in sub‐Saharan Africa; AIDS‐related KS in HIV‐positive patients; and iatrogenic KS that occurs in immunocompromised patients in the setting of long‐term immunosuppression such as in organ transplantation or in the treatment of autoimmune diseases [1, 2].

Immunosuppression results in decreased immune surveillance allowing HHV‐8 reactivation and proliferation of transformed endothelial cells. The disease most commonly manifests by involving the mucocutaneous layer, but more disseminated disease involving the viscera may also be seen [3].

Immune Thrombocytopenic Purpura (ITP) is an autoimmune disease characterized by platelet depletion due to antibody production against platelets' surface glycoproteins [4]. Treatment involves corticosteroids as first‐line therapy, and intravenous immunoglobulins (IVIG) are also considered a first‐line option, particularly in patients with acute bleeding or when a rapid increase in platelet count is required, while second‐line therapies for refractory cases include rituximab, thrombopoietin receptor agonists (TPO‐RA), and fostamatinib [5]. Rituximab is a monoclonal antibody that targets the CD20 marker on B lymphocytes, and along with glucocorticoids, it has been implicated in iatrogenic KS [6, 7].

KS in the case of ITP is rare, and has been described in only a few cases. The uniqueness of our case lies in the development of iatrogenic KS with an atypical presentation characterized by the absence of skin lesions and the presence of mucosal and visceral involvement. This rare presentation may delay diagnosis, as Kaposi sarcoma is typically associated with cutaneous manifestations that raise a flag allowing for early detection and management [8, 9, 10, 11]. Herein, we report a case of iatrogenic Kaposi sarcoma in an HIV‐negative patient with ITP who received rituximab after inadequate response to corticosteroids.

## 2 Case Presentation

A 43‐year‐old male with a known medical history of diabetes mellitus and ITP, initially diagnosed at the age of 36 years, presented with a one‐month history of cough and dyspnea. He is an ex‐smoker who quit smoking 20 years ago. He has no previous surgical history and no family history of similar conditions. Before admission, he was receiving corticosteroids and eltrombopag for ITP.

The patient was initially diagnosed with ITP at an external center and treated with oral corticosteroids (methylprednisolone 20 mg daily) for approximately 2 years, with no adequate response. At the age of 38 years, he first presented to Mansoura University Hospitals, where eltrombopag (50 mg daily) was initiated; however, platelet counts remained suboptimal. Rituximab (100 mg weekly) was subsequently started in combination with corticosteroids (20 mg daily) and eltrombopag (50 mg daily) for 6 weeks, resulting in a good hematological response. The patient was then maintained on eltrombopag (50 mg daily), with platelet counts remaining within the normal range until the age of 43 years.

At the age of 43 years, due to recurrent thrombocytopenia, rituximab was re‐initiated alongside corticosteroids and eltrombopag. After the third week of therapy, the patient developed cough and progressively worsening dyspnea over 1 month. He initially sought medical attention at a chest clinic, where he was treated empirically with antibiotics for presumed chest infection without clinical improvement. During this period, he also noticed the development of a buccal mucosal lesion, which appeared as a large brown‐colored pedunculated lesion measuring approximately 2.5 × 1.5 × 1 cm on the left lower buccal mucosa, extending to the adjacent alveolar margin, with an irregular surface, associated with ulceration and minimal bleeding. Bilateral axillary lymph node enlargement was also noted.

The patient was referred to the Oncology Center at Mansoura University Hospitals on the 26th of September 2020 the for further evaluation. Biopsies obtained from the buccal mucosal lesion and an axillary lymph node confirmed the diagnosis of Kaposi sarcoma.

His past medical history includes ITP, which was diagnosed incidentally on complete blood count (CBC). Bone marrow aspirate showed no abnormalities. Autoimmune testing, including ANA and anti‐dsDNA, as well as viral serology, were negative. He also had multiple episodes of hematemesis, during which upper gastrointestinal endoscopy revealed pedunculated gastric polyps that were biopsied and reported as hyperplastic polyps without dysplasia or atypia.

On physical examination at presentation, the patient appeared ill and in respiratory distress. Vital signs revealed tachycardia (heart rate 110 beats/min), tachypnea (respiratory rate 25 breaths/min), blood pressure of 120/80 mmHg, temperature of 37.2°C, and oxygen saturation of 80% on room air. Bilateral axillary lymphadenopathy and a buccal mucosal lesion were noted. Chest examination revealed reduced air entry and dullness to percussion on the left side. No pallor, jaundice, cyanosis, edema, or skin lesions were observed.

**FIGURE 1:**
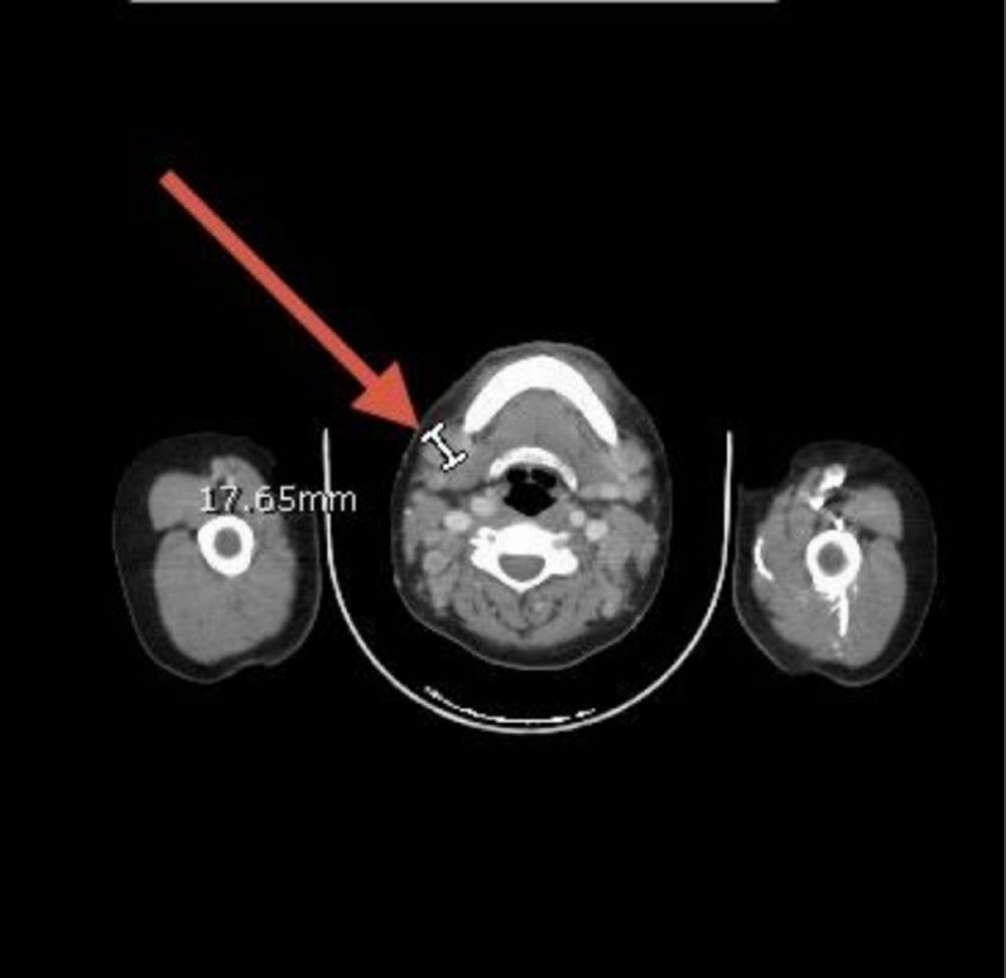
Cervical lymphadenopathy. Contrast‐enhanced CT of the neck showing multiple enlarged bilateral cervical lymph nodes across all nodal levels, consistent with pathological lymphadenopathy.

**FIGURE 2:**
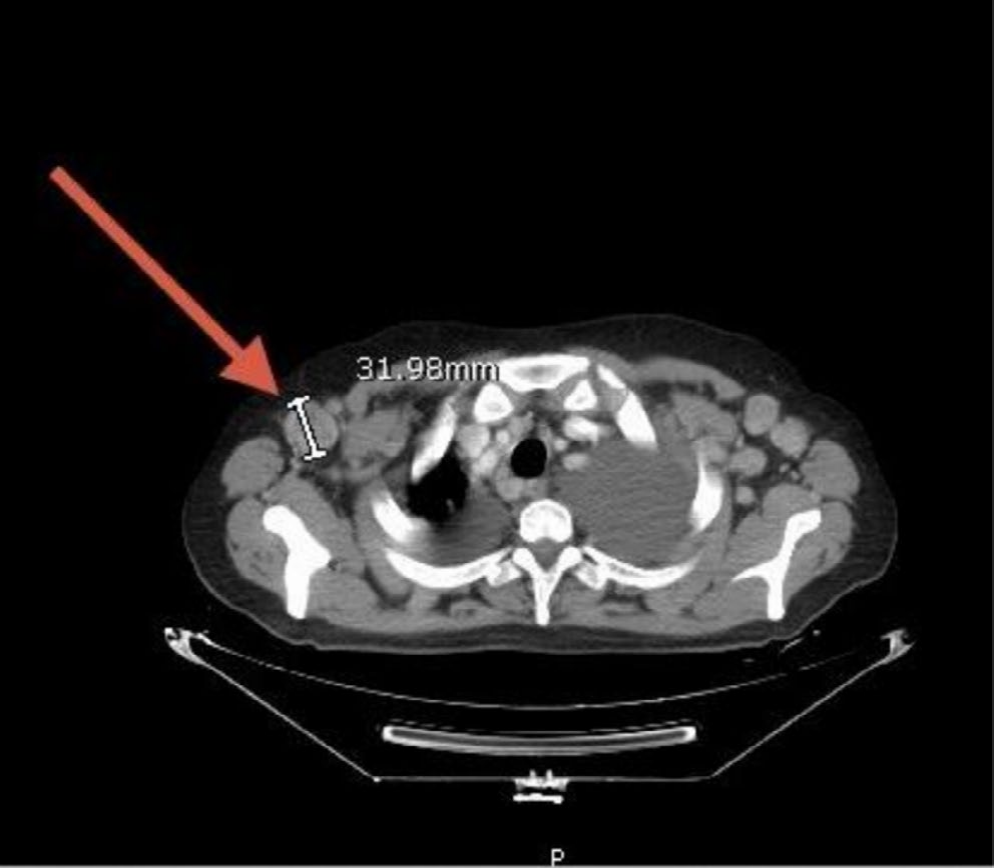
Axillary lymphadenopathy. Contrast‐enhanced CT of the axillary regions showing bilateral enlarged lymph nodes, indicating generalized nodal involvement.

**FIGURE 3:**
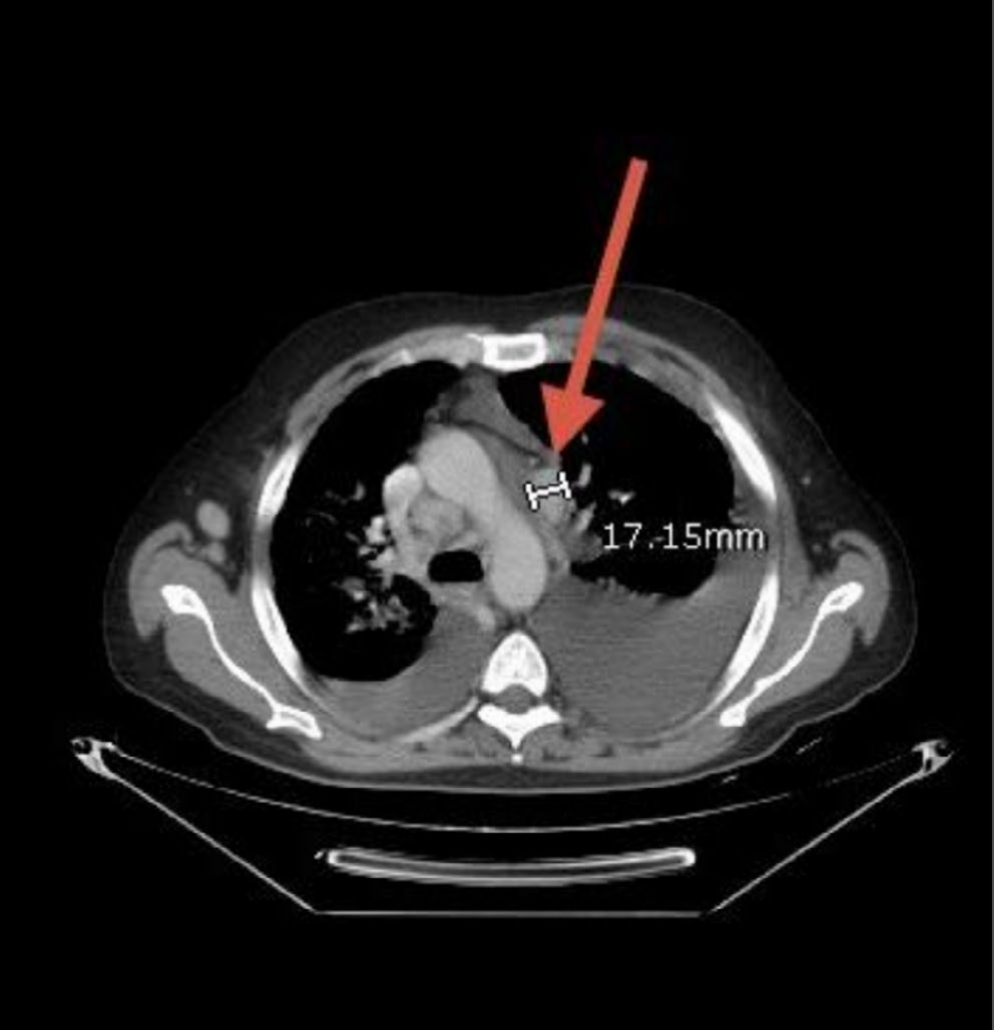
Mediastinal lymphadenopathy. Contrast‐enhanced CT showing multiple enlarged mediastinal lymph nodes, consistent with thoracic involvement in systemic disease.

**FIGURE 4:**
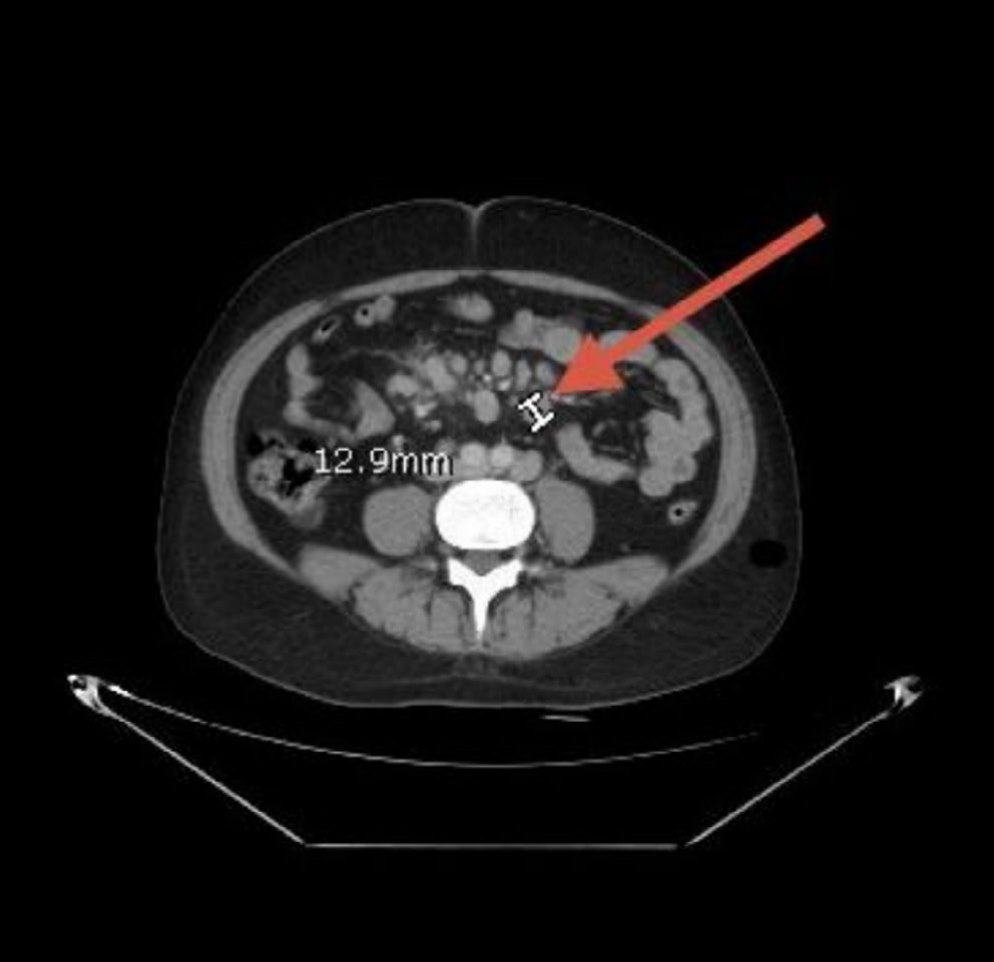
Mesenteric lymphadenopathy. Contrast‐enhanced CT of the abdomen showing enlarged mesenteric lymph nodes, reflecting intra‐abdominal nodal involvement.

**FIGURE 5:**
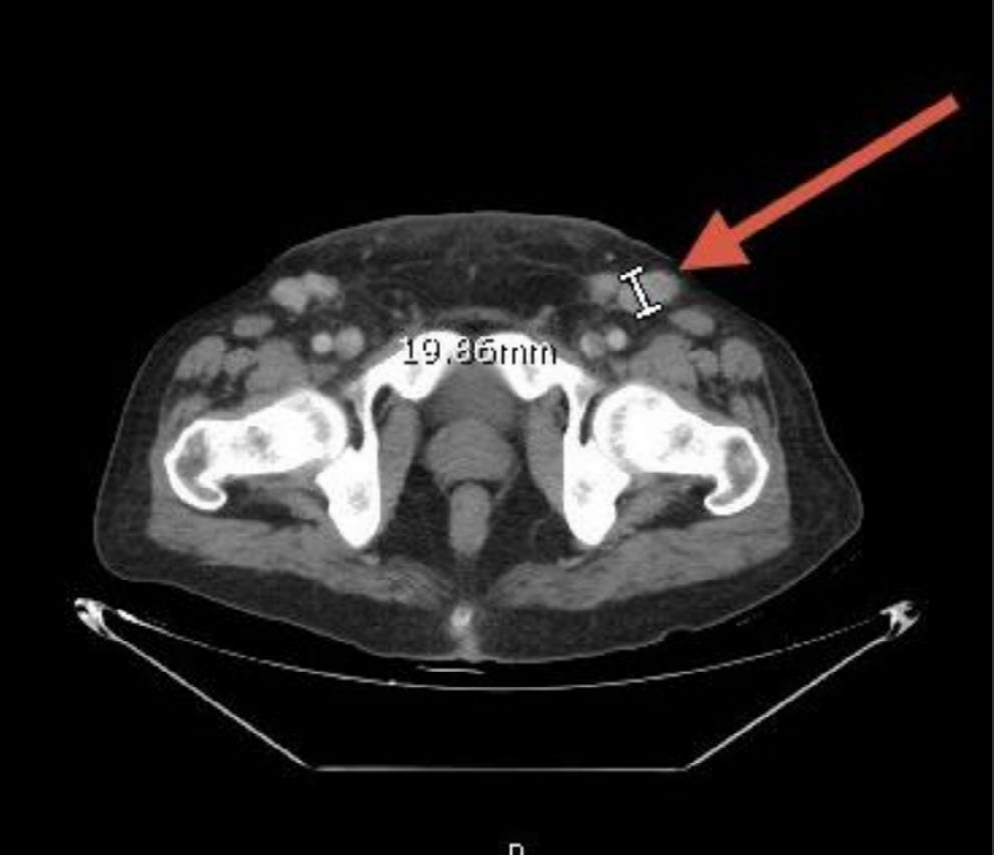
Inguinal lymphadenopathy. Contrast‐enhanced CT of the inguinal regions showing bilateral enlarged lymph nodes, supporting widespread lymphadenopathy.

**FIGURE 6:**
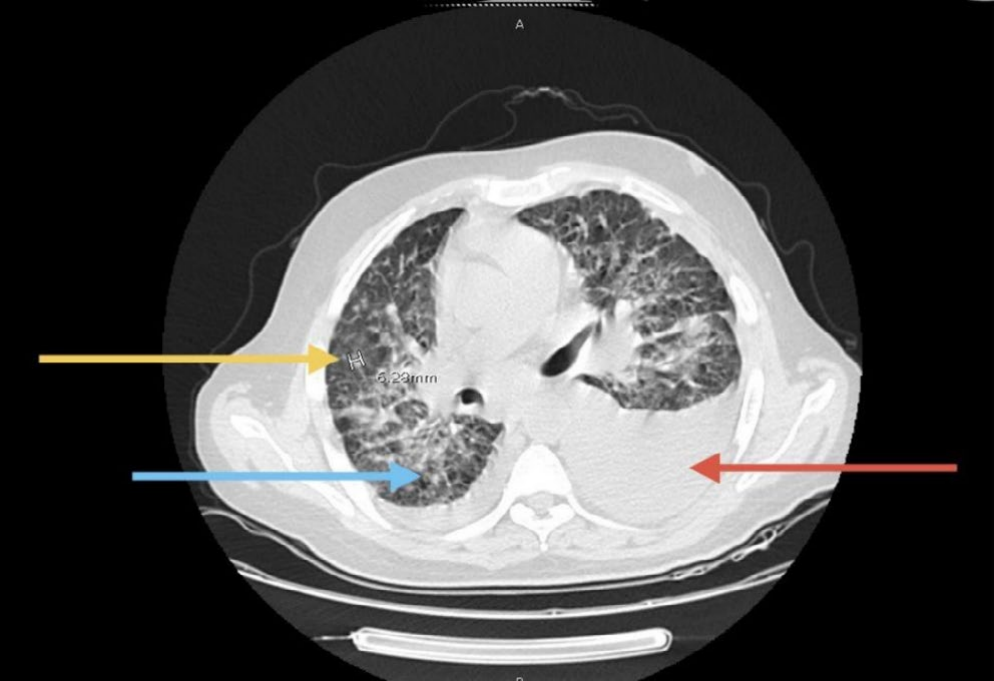
Pulmonary involvement and pleural effusion. Chest CT showing mild right‐sided and moderate left‐sided pleural effusions (red arrow), associated with bilateral ground‐glass opacities, patchy consolidation, interstitial lung thickening (blue arrow), and prominent secondary pulmonary nodules at the lung bases (yellow arrow), consistent with pulmonary dissemination of the disease.

Neck ultrasonography demonstrated multiple enlarged bilateral cervical lymph nodes, some of which lacked a detectable hilum, raising suspicion for pathological involvement. Whole‐body CT revealed generalized lymphadenopathy involving the cervical, axillary, mediastinal, mesenteric, and inguinal regions (Figures 1, 2, 3, 4, 5). Bilateral pleural effusions were present, more pronounced on the left side, along with ground‐glass opacities, consolidation patches, interstitial lung thickening, and prominent secondary pulmonary nodules at the lung bases (Figure 6). No intra‐axial or extra‐axial space‐occupying lesions were detected. There were no hepatic, splenic, pancreatic, or suprarenal deposits, and no aggressive bony lesions.

Laboratory investigations showed thrombocytopenia, leukocytosis, elevated inflammatory markers, and respiratory acidosis on arterial blood gas analysis (Table 1).

**TABLE 1:** Laboratory investigations at diagnosis.

Parameter	Result	Reference range
Hemoglobin	13.1 g/dL	13–17 g/dL
White blood cells	20.4 × 10^3^/μL	4–11 × 10^3^/μL
Platelets	83 × 10^3^/μL	150–450 × 10^3^/μL
AST (SGOT)	13.9 U/L	5–40 U/L
ALT (SGPT)	12.5 U/L	5–41 U/L
Total bilirubin	0.3 mg/dL	0.2–1.2 mg/dL
Serum creatinine	0.6 mg/dL	0.6–1.3 mg/dL
Prothrombin time	13.2 s	11–14 s
INR	1.19	0.8–1.2
PTT	32 s	25–35 s
C‐reactive protein	24 mg/L	< 5 mg/L
pH (ABG)	7.34	7.35–7.45
PaCO_2_	55 mmHg	35–45 mmHg
HCO_3_^−^	29.7 mmol/L	22–26 mmol/L

Abbreviations: ABG, arterial blood gas; INR, international normalized ratio.

The diagnosis of Kaposi sarcoma was established based on histopathological and immunohistochemical findings from the buccal mucosal lesion and axillary lymph node biopsies. Microscopic examination demonstrated a proliferation of spindle‐shaped cells with diffuse CD34 and ERG positivity. Immunohistochemical staining revealed a diffuse nuclear reaction for HHV‐8 within the spindle cells in the lymph node specimen, supporting the diagnosis. HIV testing was negative. The oncology team planned to initiate chemotherapy with either paclitaxel or doxorubicin, along with supportive care as the patient had severe respiratory symptoms and was oxygen‐dependent. He was admitted to the ward, where pleurocentesis was performed several times to relieve dyspnea.

Given the patient's poor general condition, paclitaxel was selected as the more appropriate treatment option in this clinical setting, with a weekly regimen initiated to improve tolerability. The patient was started on weekly paclitaxel at a dose of 115 mg, with marked clinical improvement after the third week of chemotherapy, including improvement in dyspnea and oxygen saturation.

However, the patient's condition deteriorated due to pulmonary hemorrhage, which may have been related to underlying ITP or paclitaxel‐induced cytopenia and increased bleeding risk. He was admitted to the intensive care unit and ultimately passed away less than 24 h after ICU admission (Table 2).

**TABLE 2:** Events timeline.

Stage	Clinical events
Initial diagnosis	Diagnosed with immune thrombocytopenic purpura (ITP) at age 36 and started on oral corticosteroids
Refractory disease	Persistent thrombocytopenia despite corticosteroid therapy over the following 2 years
Second‐line treatment	Initiation of eltrombopag 50 mg daily at age 38 at Oncology Center, Mansoura University Hospitals
Combination therapy	Addition of rituximab 100 mg weekly for 6 weeks with good hematological response
Stable disease	Maintained on eltrombopag with stable platelet counts until age 43
Relapse	Recurrence of thrombocytopenia at age 43; rituximab re‐initiated with corticosteroids and eltrombopag
Symptom development	Development of cough and progressive dyspnea after the third dose of rituximab
Initial misdiagnosis	Treated as chest infection without improvement
Disease progression	Development of buccal mucosal lesion and bilateral axillary lymphadenopathy
Diagnosis	Biopsy confirmed Kaposi sarcoma HHV‐8 positive, HIV negative; CT showed disseminated disease
Treatment	Initiation of weekly paclitaxel 115 mg
Clinical response	Improvement after 3 weeks with better dyspnea and oxygenation
Complication	Development of pulmonary hemorrhage secondary to ITP
Outcome	ICU admission followed by death within 24 h

This case represents a rare iatrogenic Kaposi sarcoma in an HIV‐negative patient receiving corticosteroids and rituximab for ITP, with confirmed HHV‐8 positivity. The disease presented exclusively with extracutaneous involvement of the lungs, buccal mucosa, and lymph nodes, without skin lesions.

## 3 Discussion

Kaposi sarcoma (KS) is clinically classified into four major variants: classic KS, endemic African KS, HIV‐associated KS, and iatrogenic KS [2]. The disease is strongly linked to infection with HHV‐8 [12]. However, clinical disease usually emerges when immune function is compromised, such as in the setting of HIV infection, organ transplantation, or prolonged exposure to immunosuppressive therapy [2]. The iatrogenic variant of KS is relatively uncommon and has been reported to account for approximately 5%–20% of cases in published series [13, 14, 15]. This subtype was first described among organ transplant recipients receiving long‐term immunosuppressive therapy [16]. It has also been observed in patients receiving immunosuppressive therapy for non‐transplant indications, such as corticosteroids and rituximab [3, 7, 17, 18].

Only a small number of reports have described KS in association with ITP [8, 9, 10, 11]. In most of these cases, the development of KS appears to be related to immunosuppressive therapy, particularly corticosteroids or rituximab, rather than to ITP itself. This association may be explained by the immunomodulatory effects of these drugs. Rituximab may promote the development of KS by causing prolonged depletion of B lymphocytes, which impairs immune surveillance against HHV‐8 and may allow viral reactivation [19]. It may also lead to a decrease in T‐cell activation, which could further promote HHV‐8 reactivation [20]. However, data on B‐cell counts and immunoglobulin levels following rituximab therapy were not available, as these investigations are not routinely performed at Mansoura University Hospitals, and treatment response was assessed based on platelet count trends and overall clinical improvement. Corticosteroids may also promote the development of KS by stimulating KS cell proliferation through inhibition of transforming growth factor‐β, which normally suppresses endothelial growth, and by increasing the expression of glucocorticoid receptors on KS cells [6, 21]. Notably, the patient had received prolonged oral corticosteroid therapy (methylprednisolone 20 mg daily) for approximately two years prior to presentation at the same center, with no adequate response. This extended duration of therapy was initiated and continued at an external center, where the rationale for continuing this therapy was not documented.

In our HIV‐negative patient, the occurrence of KS while receiving both rituximab and corticosteroids suggests that a treatment‐related mechanism is the most plausible explanation. The most significant aspect of this case is the occurrence of disseminated KS without cutaneous involvement, dominated by pulmonary and nodal disease. Previous reports in HIV‐negative ITP patients developed only cutaneous KS nodules that regressed after steroid tapering and chemotherapy administration [8], or with steroid tapering alone [9, 10, 11]. Notably, these cases involved immunosuppression with corticosteroids alone, whereas our patient was exposed to prolonged corticosteroid therapy followed by combined treatment with rituximab and corticosteroids. The onset of symptoms shortly after re‐exposure to rituximab suggests a temporal association, consistent with a prior case describing a shorter interval to KS development in patients receiving rituximab‐based therapy compared with corticosteroids alone [22].

The non‐specific presentation seen in our patient has led to a delay in diagnosis as the respiratory symptoms of pulmonary KS may overlap with other opportunistic infections [23], thus, the patient was initially managed as a case of chest infection until buccal lesion and axillary lymph node enlargement occurred.

Definitive diagnosis was established through biopsy, which confirmed KS by histological examination and immunohistochemical staining for HHV‐8 antigens. Histopathology remains the cornerstone of KS diagnosis and is largely consistent across subtypes, typically demonstrating spindle‐shaped cells forming irregular vascular spaces with erythrocyte extravasation and a variable inflammatory infiltrate [24, 25]. Because these features may overlap with other vascular tumors, immunostaining for HHV‐8 latency‐associated nuclear antigen (LANA) is essential for diagnostic confirmation [26].

Management of KS depends on disease subtype, extent, and patient condition. Although no standardized guidelines exist specifically for iatrogenic KS, disseminated disease is generally treated with systemic chemotherapy using pegylated liposomal doxorubicin or paclitaxel as first‐line agents [27]. Concurrently, reduction or withdrawal of immunosuppressive therapy is recommended where feasible [28]. In patients with ITP, however, this approach poses a significant therapeutic dilemma, as decreasing immunosuppression while administering chemotherapy may precipitate severe thrombocytopenia and bleeding, whereas continued immunosuppression risks progression of KS [29]. In our patient, paclitaxel was appropriately initiated and resulted in clinical improvement; nevertheless, fatal pulmonary hemorrhage occurred as either a complication of underlying ITP or due to treatment with paclitaxel.

The prognosis largely depends on the ability to reduce immunosuppression [27], and on the extent of visceral involvement [30]. Disseminated disease, particularly with pulmonary or lymph node involvement, is associated with a more aggressive clinical course and poorer outcomes compared with isolated cutaneous lesions. In our case, the outcome was unfavorable due to complications related to severe thrombocytopenia rather than direct tumor progression.

This case highlights an uncommon presentation of iatrogenic Kaposi sarcoma in an HIV‐negative patient receiving rituximab and corticosteroids for ITP, with exclusively extracutaneous involvement leading to diagnostic delay. It emphasizes the importance of maintaining a high index of suspicion in immunosuppressed patients with unexplained respiratory or mucosal symptoms, even without skin lesions, and the importance of early histopathological confirmation to guide management. It also illustrates the therapeutic challenge of balancing immunosuppression and bleeding risk in patients with concomitant ITP, emphasizing the need for a multidisciplinary approach to improve outcomes.

A limitation of this report is the unavailability of representative histopathological images, as the histopathological and immunohistochemical analyses were performed at an external laboratory and access to the original images was not possible. In addition, HHV‐8 testing was not performed on peripheral blood or pleural fluid.

## 4 Conclusion

This is a case of an uncommon but serious form of iatrogenic Kaposi sarcoma in an HIV‐negative patient with ITP receiving rituximab and corticosteroids. The disease manifested solely as extracutaneous involvement with pulmonary, mucosal, and lymph node disease in the absence of skin lesions, which resulted in a delay in diagnosis. Chemotherapy was associated with partial clinical response; however, in the setting of disseminated Kaposi sarcoma and concomitant immune‐mediated thrombocytopenia, management decisions became complicated, leading to fatal hemorrhage. Clinicians must maintain a high index of suspicion for Kaposi sarcoma in immunocompromised HIV‐negative individuals with unexplained respiratory or mucosal involvement. Early histologic confirmation is important, as early diagnosis could improve patient management and outcomes in an immunocompromised population.

## Author Contributions

**Mohammed Yousef Awad:** writing – original draft, writing – review and editing. **Esraa Elhakim:** writing – original draft, writing – review and editing. **Rami Khader:** writing – original draft, writing – review and editing. **Mousa Almasalma:** writing – original draft, writing – review and editing. **Ameed Khader:** writing – original draft, writing – review and editing. **Ro'a Draidi:** writing – original draft, writing – review and editing. **Ayman Zebda:** writing – original draft, writing – review and editing. **Mohammad Bdair:** writing – original draft, writing – review and editing.

## Funding

The authors have nothing to report.

## Ethics Statement

The authors have nothing to report.

## Consent

Written informed consent was obtained from the patient for their anonymized information to be published in this article.

## Conflicts of Interest

The authors declare no conflicts of interest.

## Data Availability

Data sharing not applicable to this article as no datasets were generated or analyzed during the current study.
